# Primary cutaneous non neuroendocrine small cell carcinoma POU2F3 subtype: morphologic, immunohistochemical, transcriptomic and methylation analysis of two cases

**DOI:** 10.1007/s00428-026-04406-4

**Published:** 2026-01-22

**Authors:** Kerman Zyani, Daniel Pissaloux, Charly Liddell, Mahtab Samimi, Franck Tirode, Andreas von Deimling, Serge Guyétant, Sylvie Lantuejoul, Thibault Kervarrec

**Affiliations:** 1 Department of Pathology, Université de Tours, Centre Hospitalier Universitaire de Tours, Tours, France; 2 Department of Biopathology, Centre Léon Bérard, Centre de Recherche en Cancérologie de Lyon, INSERM U1052 - CNRS UMR5286, Université Claude Bernard Lyon 1, Lyon, France; 3 Department of Pathology, Hospital of La Roche Sur Yon, La Roche-sur-Yon, France; 4 Department of Dermatology, Université de Tours, Centre Hospitalier Universitaire de Tours, Tours, France; 5 “Biologie Des Infections À Polyomavirus” Team, UMR INRA ISP 1282, Université de Tours, Tours, France; 6 CARADERM Network, Lille, France; 7 Department of Neuropathology, Institute of Pathology, Ruprecht-Karls-University, Heidelberg, Germany; 8 Clinical Cooperation Unit Neuropathology, German Cancer Research Center (DKFZ), and German Cancer Consortium (DKTK), Heidelberg, Germany; 9 Grenoble Alpes University, Grenoble, France

**Keywords:** Carcinoma, Neuroendocrine differentiation, POU2F3 subtype

## Abstract

Up to 10% of small cell lung carcinomas do not express any neuroendocrine markers and are characterized by an expression of the transcription factor POU2F3. Recently, few cases of poorly differentiated carcinomas harboring POU2F3 expression have been described in the breast, cervix, and bladder. Herein, we report the morphological, immunohistochemical, and molecular characterization of two primary cutaneous POU2F3-expressing small cell carcinomas. The cases arose in the temple and on the occipital region in a 92-year-old woman and a 78-year-old man respectively. Microscopic examination revealed in both cases a large and ulcerated poorly differentiated neoplasm infiltrating the full thickness of the dermis with extension into the subcutaneous tissues in case #1. The tumors harbored solid and trabecular growth patterns and were composed of poorly differentiated highly mitotic cells. Immunohistochemical investigation revealed diffuse pancytokeratin positivity while no expression of cytokeratin 20, chromogranin A, synaptophysin, CD56, or INSM1 was detected. Diffuse nuclear expression of POU2F3 was observed. Transcriptomic analysis revealed a SBS7 mutation signature related to UV-induced DNA damages in one case and evidenced an expression profile similar to the one observed in POU2F3-expressing small cell lung cancers in both. Methylation analysis confirmed the proximity of the two cases with other skin cancers. To conclude, we report herein the first description of primary cutaneous small cell carcinoma POU2F3 subtype.

**Supplementary Information:**

The online version contains supplementary material available at https://doi.org/10.1007/s00428-026-04406-4.

## Introduction

Neuroendocrine neoplasms may arise in virtually all anatomical locations and are subclassified into well-differentiated/low-grade neuroendocrine tumors and poorly differentiated/high-grade carcinomas [1]. In the skin, Merkel cell carcinoma (MCC) is defined according to the current WHO classification as the eponymous name for primary cutaneous neuroendocrine carcinoma [2]. In the lung, high-grade neuroendocrine carcinomas are categorized into large cell and small cell types. In this latter group, up to 10% of the cases do not express the neuroendocrine markers and harbor an expression profile similar to the physiological tuft cells notably with the expression of the transcription factor POU2F3 [3–5].

Recently, few cases of poorly differentiated carcinomas harboring POU2F3 expression without neuroendocrine differentiation, reported as “tuft cell carcinomas,” have been described in the breast, cervix, and bladder [6]. To our knowledge, no tumor harboring such features has been described in the skin so far.

Here, we report morphological, immunohistochemical, and molecular characterization of two cases of POU2F3-expressing non-neuroendocrine small cell carcinoma of the skin.

## Methods

### Case selection

Cases of high-grade poorly differentiated cutaneous carcinoma with POU2F3 expression were identified from the consultation files of the authors (CL, SG, and TK). To ensure the exclusion of skin metastasis, only cases with no prior history of cancers, with a negative metastatic work-up, or evidence of UV signature after molecular analysis were included in the study. For the methylation study, control cases included 37 small cell lung cancers, 41 Merkel cell carcinomas, 11 cutaneous squamous cell carcinomas, and 9 basal cell carcinoma cases from the Heidelberg University database. The design of this retrospective study was in accordance with the requirements for the use of biological material in research proposed by the ethical guidelines of our institution (Local Ethics Committee, Tours, France; No. ID RCB2009-A01056-51). The following clinical data were collected from the patient files: sex, age, tumor location and tumor size, follow-up.

### Immunohistochemistry

Immunohistochemical staining for cytokeratin AE1/AE3, BAP1, BCL2, CD117, CD56, chromogranin A, CK18, CK5/6, CK7, CK20, CMYC, INSM1, ISL1, KI67, neurofilament, p40, POU2F3, POU4F3, synaptophysin, SOX2, and SOX9 was performed using a Ventana BenchMark XT Platform (Roche Diagnostics, Basel, Switzerland) as instructed. Dilution and clones are available in Supplementary Table 1.

### RNA sequencing

RNA sequencing was performed in all cases as previously described [7]. Briefly, total RNA was extracted from FFPE tissue sections. For each sample, 100 ng of total RNA was used to prepare the library with the TruSeq RNA Access Library Prep Kit (Illumina, San Diego, CA, USA). Fourteen libraries were pooled at 4 nM with PhiX Control in a low-concentration spike-in (1%). Sequencing was performed (75 cycles paired-end) with NextSeq 500/550 High Output V2 Kit in NextSeq 500 (Illumina). The samples were analyzed with the BaseSpace sequence Hub (Illumina) with RNA-Sequencing alignment application. The alignments were realized with Star and TopHat2 on the GRCh37 reference genome. The fusion transcript was called with Manta, EricScript, and TopHat2 fusion.

### Analysis of DNA methylation

Genomic DNA was extracted from formalin-fixed paraffin-embedded tissues using the Maxwell apparatus (Promega, Madison, WI, USA) with Maxwell 16 FFPE Plus LEV DNA Purification kits. Methylation profiling employed the MethylationEPIC BeadChip (Illumina, San Diego, SA, USA). Three hundred nanograms of DNA was processed according to the manufacturer’s protocol as previously described [8]. Data were generated in the Department of Neuropathology of the University Hospital Heidelberg. Computational analyses were based on R version 4.6.1 (https://www.R-project.org). t-SNE plot was generated on 10,000 most variable CpG sites upon standard deviation, 3000 iterations, and using a perplexity parameter of 10.

## Results

### Case #1

**Fig. 1:**
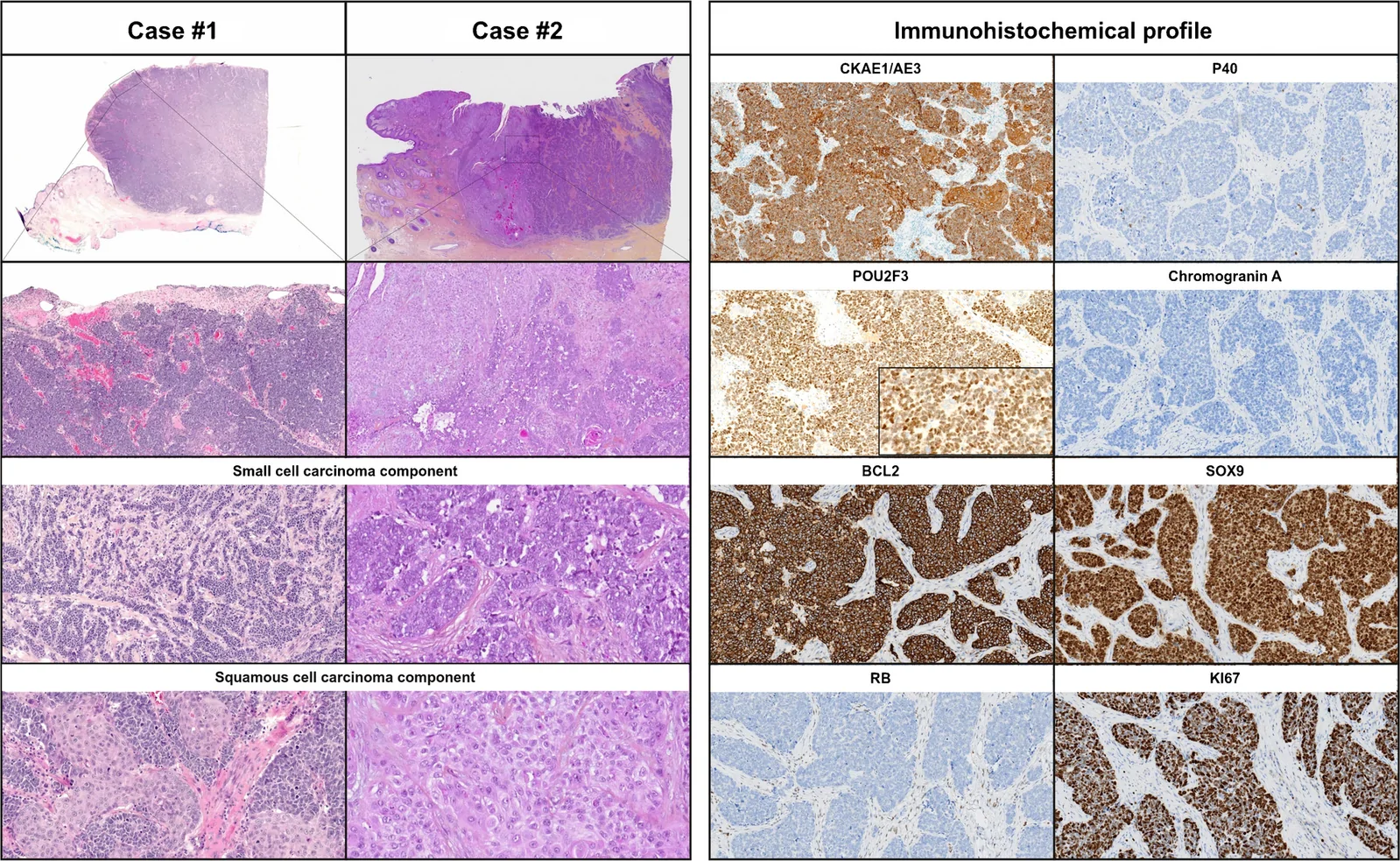
Morphological and immunohistochemical features of two POU2F3-expressing carcinomas of the skin. Microscopic examination revealed in both cases a large and ulcerated poorly differentiated neoplasm infiltrating the full thickness of the dermis with extension into the subcutaneous tissues in case #1. The tumor harbored solid and trabecular growth patterns and was composed of poorly differentiated highly mitotic cells. In both cases, a squamous cell carcinoma component was present in association with the main POU2F3-expressing part. Immunohistochemical investigation revealed diffuse pancytokeratin positivity while P40 was restricted to the squamous cell carcinoma component. No expression of chromogranin A was detected. Nuclear POU2F3 expression was evidenced in the basophilic component in association with BCL2 and SOX9 positivity. RB was lost. High proliferation index was evidenced (ki67 staining)

A 92-year-old non-smoker woman with no prior history of cancer was referred to a plastic surgeon for a fast-growing nodular tumor located on the right temple (Fig. 1). Clinical examination revealed a well-demarcated, erythematous, ulcerated nodule measuring 4 cm.

Microscopic examination of the tumor mass (Fig. 1) revealed an ulcerated, poorly differentiated neoplasm infiltrating the full thickness of the dermis and extending into the subcutaneous tissues. The tumor harbored solid and trabecular growth patterns and was composed of basophilic cells with high nucleocytoplasmic ratios, round to oval medium-sized nuclei, vesicular chromatin. Frequent nuclear molding and numerous mitotic figures were present. Focally, a minor component of squamous cell carcinoma was detected in the superficial part of the tumor intermixed with the basophilic part. Vascular invasion was noted.

Immunohistochemical investigation of the case revealed diffuse pancytokeratin positivity (AE1/AE3) and heterogeneous cytokeratin 7 expression. P40 was only expressed in the squamous component. Loss of RB1 protein expression was evidenced in both tumor parts. No expression of cytokeratin 20, chromogranin A, synaptophysin, CD56, INSM1, ISL1, SOX2, neurofilament, or POU4F3 was detected. The proliferation index (Ki-67) was up to 90%. Diffuse strong nuclear expression of POU2F3 was observed, along with expression of other tuft cell markers, i.e., SOX9, BCL2, CD117, and CK18 (Fig. 1). CMYC was expressed in approximately 70% of tumor cells. Loss of BAP1 expression was also noted.

Next-generation sequencing revealed a high tumor mutational burden (TMB = 18,09/Mb) with a prominent SBS7 cosmic mutation signature (Supplementary Fig. 1) consistent with ultraviolet radiation–induced DNA damage. *TP53*.pR249G, *TP53*.pR342*, *RB1*.pQ257*, *FGFR3*.pS408F, and *BAP1*.pL100Pfs* were identified among the pathogenic variants. In this context, a diagnosis of primary cutaneous non-neuroendocrine small cell carcinoma POU2F3 subtype was made. The patient died from heart failure 3 months after the diagnosis.

### Case #2

A large and ulcerated tumor mass of the occipital region was excised in a 78-year-old man, active smoker (15 cigarettes/day). The patient described a long-standing lesion, which accelerated tumor growth following a cranial trauma 1 year before. Extension work-up (CT and PET) demonstrated laterocervical and occipital lymphadenopathy without distant tumor mass.

Gross examination of the excised specimen revealed a large polypoid lesion, measuring 6 × 4.5 cm. Histological examination showed a biphasic tumor infiltrating the full thickness of the dermis without subcutaneous involvement (Fig. 1). At the periphery of the tumor mass, a mostly in situ keratinizing squamous cell carcinoma component was present composed of large eosinophilic cells with atypical nuclei. An abrupt transition was observed between this area and the main tumor component characterized by nested and trabecular growth patterns and composed of poorly differentiated basophilic, small-to-medium-sized cells with scant cytoplasm and oval nuclei. Vascular invasion was observed.

Immunohistochemistry demonstrated diffuse p40 positivity restricted to the squamous component. Neuroendocrine markers (chromogranin A, synaptophysin, CD56, INSM1, neurofilament) were negative as well as cytokeratin 20. KI67 staining revealed a proliferation index up to 70%. RB1 loss was restricted to the basophilic component. In this later, diffuse nuclear POU2F3 expression was detected associated with BCL2, CMYC, SOX9, and CD117 positivity.

Molecular analysis revealed *RB1* pQ395* and *TP53* pP77Sfs*70 pathogenic variants in the basophilic part.

These features supported the diagnosis of a primary cutaneous non-neuroendocrine small cell carcinoma POU2F3 subtype. No evidence of recurrence was detected after a 4-month follow-up.

### Molecular characterization of the cases

To further characterize primary cutaneous POU2F3-expressing non-neuroendocrine carcinoma as a distinctive tumor entity, the two cases were submitted to methylation and transcriptomic analysis as previously described [7, 9] using a control series including skin and lung tumors.

**Fig. 2:**
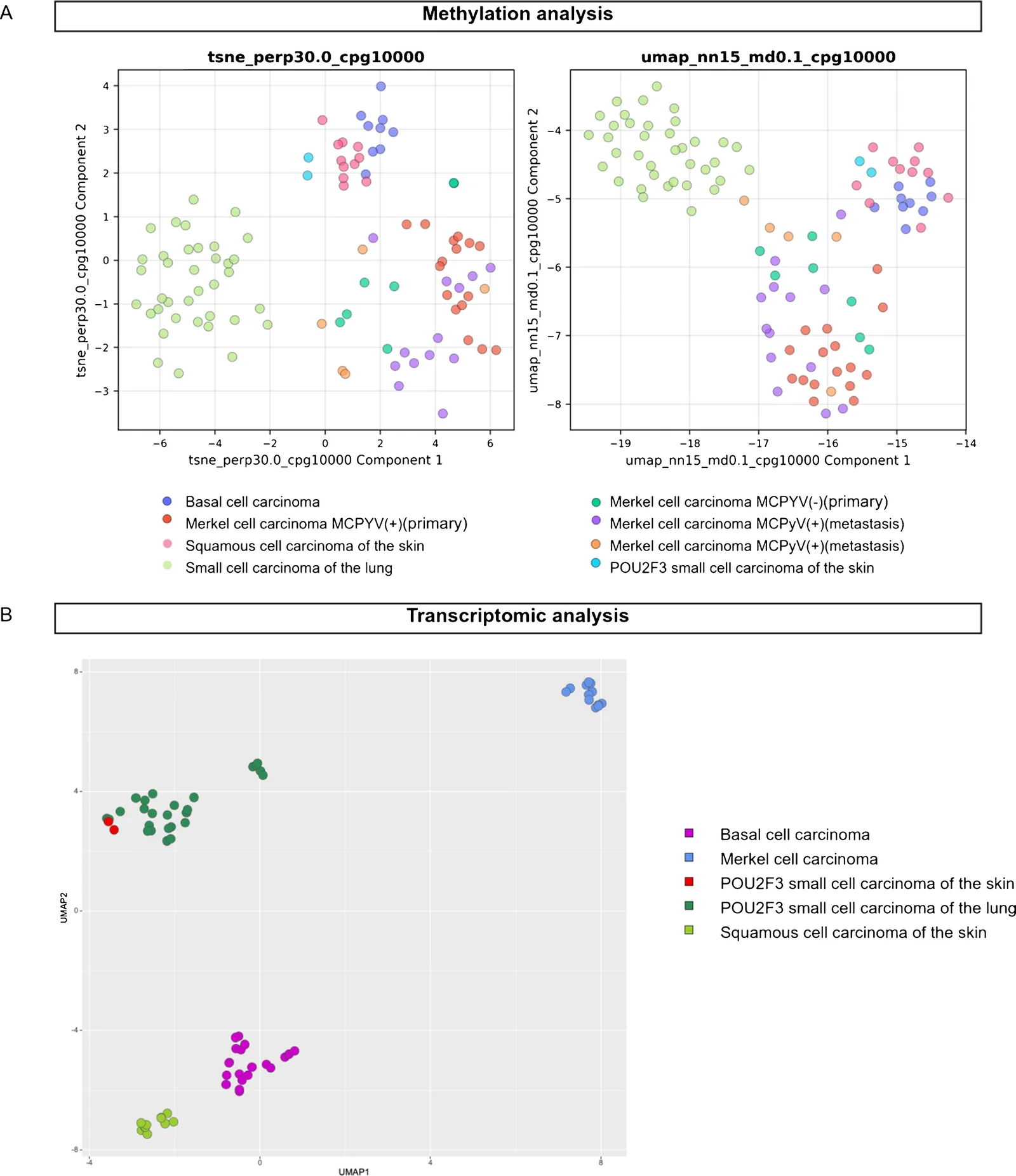
Molecular features of the cases. **A** Methylation analysis. A UMAP and a t-SNE analysis are depicted. These analyses revealed that primary cutaneous POU2F3-expressing carcinoma harbors a similar profile, close to the cutaneous squamous cell carcinoma and distinct from the lung tumors. **B** Transcriptomic analysis. A UMAP is depicted. Such analysis confirmed that the expression profile of primary cutaneous POU2F3-expressing cases is similar to the one observed for small cell carcinoma POU2F3 variant arising in other organs and distinct from skin tumors especially Merkel cell carcinoma

Indeed, the methylation profile is expected to be preserved during the oncogenesis and therefore might be used to identify tumor origin [9]. In this context, our two cases were compared to 37 small cell lung cancers, 41 Merkel cell carcinomas, 11 cutaneous squamous cell carcinomas, and 9 basal cell carcinoma cases. Results are available in Fig. 2 (UMAP and t-SNE analysis). Such analysis revealed that the two primary cutaneous POU2F3-expressing cases harbored similar methylation profiles, distinct from that of small cell lung cancers and close to that of cutaneous squamous cell carcinoma.

The transcriptomic profile of the cases was then performed in comparison to small cell lung cancers—including POU2F3 subtype—as well as cutaneous tumors. The UMAP is available in Fig. 2. By contrast to the methylation features, this analysis demonstrated that POU2F3-expressing cutaneous tumors harbored an expression profile similar to POU2F3-expressing cases arising from other organs and separate from other primary cutaneous tumors, including Merkel cell carcinoma.

## Discussion

Small cell lung carcinomas (SCLC) are currently stratified into transcriptomic subtypes—ASCL1, NEUROD1, YAP1, and POU2F3—each defined by a dominant transcription factor with distinct transcriptomic landscapes [3, 5, 10]. While ASCL1 and NEUROD1 are the most frequent subtypes, associated with neuroendocrine differentiation and classic small cell morphology, the POU2F3 variant lacks neuroendocrine differentiation [5]. To note, the existence of the YAP1 subtype has been challenged by recent studies suggesting that YAP1 expression may be a variable feature associated with non-neuroendocrine or inflamed tumor phenotypes, rather than representing a true lineage-specific entity [11–15].

By contrast, POU2F3 defines a non-neuroendocrine lineage [5] with phenotypic similarities with the tuft cells [16], a rare population of epithelial chemosensory cells first identified in the gastrointestinal tract and airways [17–19]. These cells express *POU2F3* as well as *ASCL2* and *SOX9*, and are involved in sensing environmental signals and immune responses regulation [17–19]. In mouse models, POU2F3 is required for physiologic tuft cell development and homeostasis [18]. Accordingly, in tumors, experimental models have demonstrated that *POU2F3*-expressing SCLCs are transcriptionally dependent on this transcription factor and demonstrate distinct therapeutic vulnerabilities, including sensitivity to IGFR inhibitors [16]. While better outcome was demonstrated in this setting in comparison to neuroendocrine subtypes of SCLC in some studies [20, 21], these findings remain controversial [10]. Microscopically, POU2F3-expressing SCLC seems to have overlapping features between small cell and large cell neuroendocrine carcinoma, have more abundant tumor stroma and more prominent nest formation than the other SCLC subtypes, and are frequently combined with squamous cell carcinomas [20]. Interestingly, genetic comparison of POU2F3 subtype with other SCLC revealed more frequent *PTEN* inactivation*, MYC* amplifications, and 20q13 amplifications in the POU2F3 subgroup [20].

Beyond lung cancers, POU2F3-positive small cell carcinomas have been reported in a limited number of extrapulmonary sites such as the breast, cervix, and bladder [6]. These tumors consistently lack neuroendocrine marker expression and mirror the tuft cell–like transcriptomic profile of their pulmonary counterparts [6]. The specificity of POU2F3 as a lineage marker across organs is thus increasingly supported. In a recent large-scale study by Yamada et al., among 6064 carcinomas (including 39 basal cell carcinomas, 27 Merkel cell carcinomas, and 32 squamous cell carcinomas of the skin) and 2730 mesenchymal neoplasms, a total of only 43 tumors (0.4%) demonstrated POU2F3 positivity [22]. These included one Merkel cell carcinoma and two basal cell carcinomas, further highlighting the rarity of POU2F3 expression in cutaneous tumors and supporting the exceptional nature of our cases. Accordingly, by investigating a previously described cohort including 165 cutaneous MCCs [23], we did not identify any case with POU2F3 expression (data not shown). Interestingly, a previous transcriptomic comparison of Merkel cell carcinoma with small cell lung cancers suggested that at least a part of the Merkel cell carcinoma expressed NEUROD1 [24, 25] while ASCL1 was not observed. Furthermore, POU4F3, another member of the POU transcription factor family, was recently identified as a sensitive and specific diagnosis marker of MCC [26], such marker being not expressed in our two POU2F4-expressing cases.

In both of our cases, a minor component of squamous cell carcinoma was detected in the superficial part of the tumor. Although a focal metaplastic squamous differentiation of small cell carcinoma POU2F3 subtype may be discussed, this finding may alternatively suggest the squamous cell carcinoma as a precursor of the POU2F3-expressing component, according to a transformation sequence similar to that observed in a subset of MCC, especially the one not related to polyomavirus integration [27–30]. From the genetic level, our two POU2F3-expressing tumors seem to share with MCC [31] similar genetic alterations including inactivation of *RB1* and *TP53* pathways. However, as observed in lung tumors [12], we detected overexpression of *CMYC* in POU2F3-expressing skin carcinomas, *CMYC* amplification being recurrent in cutaneous squamous cell carcinoma [32] but lacking in MCC harboring *LMYC* amplification [33]. Therefore, it is possible that in a common precursor, i.e., squamous cell carcinoma, in addition to *TP53* and *RB1* inhibitions, additional and mutually exclusive genetic events including *CMYC* or *LMYC* amplification drive the transformation into either POU2F3-expressing carcinomas or Merkel cell carcinoma, as already described in lung cancers[34].

One limitation of our study is the lack of extension work-up in case #1 due to the advanced age and comorbidity of the patient, who died quickly after the diagnosis. However, the patient was a non-smoker with no prior history of cancers and molecular analysis confirmed a UV-induced mutational signature while the methylation profile indicated a cutaneous origin and ruled out a skin metastasis from a SCLC.

To conclude, we report for the first time to our knowledge the clinical, morphological, immunohistochemical, transcriptomic, and methylation description of two primary cutaneous carcinomas POU2F3 subtype. This emerging tumor entity should be considered in the differential diagnosis of cutaneous basaloid neoplasms with high-grade features but lacking neuroendocrine differentiation.

## Supplementary Information

Below is the link to the electronic supplementary material.

ESM1 (DOCX.102 KB)
