# Supplementary material for: Primary cutaneous non neuroendocrine small cell carcinoma POU2F3 subtype: morphologic, immunohistochemical, transcriptomic and methylation analysis of two cases
Source: Virchows Arch. 2026 Jan 22;489(4):1005–11. doi: 10.1007/s00428-026-04406-4 (PMC13714156; doi:10.1007/s00428-026-04406-4)
Supplement: Supplementary file 1 [file 428_2026_4406_MOESM1_ESM.docx]

**Supplements**

**Supplementary Table 1. Antibodies used for immunohistochemistry.**

**Supplementary Figure 1.** **Mutational profile of the case #1.** Genetic studies revealed a genomic profile with a predominance of C to T transitions and close to the SBS7B signature evocative of DNA damages induced by UV exposure.

**Supplementary Table 1. Antibodies used for Immunohistochemistry.**

| **antigen** | **clone** | **company** | **dilution** |
| --- | --- | --- | --- |
| BAP1 | C-4 | Diagomics | 1/50 |
| BCL2 | MO887 | Dako | 1/50 |
| CD56 | MRQ 42 | Cell Marque, Rocklin, California, USA | Pre-diluted |
| CD117 | Polyclonal | Dako | 1/300 |
| Chromogranin A | A0430 | Dako | 1/2000 |
| CKAE1/AE3 | M3515 | Dako, Glostrup, Denmark | 1/200 |
| CK7 | M7018 | Dako | 1/200 |
| CK18 | DC10 | Dako | Pre-diluted |
| CK20 | Ks20.8 | Dako | 1/100 |
| CMYC | Y69 | Ventana | Pre-diluted |
| INSM1 | A-8 | Clinisciences | 1/150 |
| ISL1 | EP283 | BioSB, | Pre-diluted |
| Ki67 | 30-9 | Ventana, | Pre-diluted |
| Neurofilament | 2F11 | Dako | Pre-diluted |
| P40 | polyclonal | Zytomed | 1/200 |
| POU2F3 | polyclonal | Invitrogen | 1/100 |
| POU4F3 | QQ8 | SantaCruz | 1/100 |
| RB1 | 13A10 | Leica | 1/50 |
| Synaptophysin | RBK011 | Dako | 1/200 |
| SOX2 | SP76 | Cell Marque | Pre-diluted |
| SOX9 | AB5535 | Merck | 1/1000 |

**Supplementary Figure 1.** **Mutational profile of the case #1.** Genetic studies revealed a genomic profile with a predominance of C to T transitions and close to the SBS7B signature evocative of DNA damages induced by UV exposure.
